# Teen Dating Violence, Sexism, and Resilience: A Multivariate Analysis

**DOI:** 10.3390/ijerph17082652

**Published:** 2020-04-13

**Authors:** María Dosil, Joana Jaureguizar, Elena Bernaras, Juliana Burges Sbicigo

**Affiliations:** 1 Research and Diagnosis Methods in Education, Faculty of Education, University of Basque Country, 48940 Lejona, Spain; maria.dosil@ehu.eus; 2 Developmental and Educational Psychology Department, Faculty of Education of Bilbao, University of the Basque Country, 48940 Lejona, Spain; 3 Developmental and Educational Psychology Department, Faculty of Education, Philosophy and Anthropology, University of the Basque Country, 20018 Donostia, Spain; elena.bernaras@ehu.eus; 4 Developmental and Personality Psychology Department, Universidade Federal do Rio Grande do Sul, 90035-003-Porto Alegre-RS, Brasil; juliana.sbicigo@ufrgs.br

**Keywords:** teen dating violence, adolescents, predictors, adjustment, sexism, resilience

## Abstract

The aim of this research was to know the factors associated with teen dating violence and victimization because violence in teenagers’ relationships is increasing in recent years, constituting a serious social problem. For this purpose, we analyzed teen dating violence and explored the variables (sexist attitudes, personal adjustment, clinical maladjustment, and resilience) related to teen dating violence and victimization using multinomial logistic models. The sample was composed of 268 school teenagers aged 12 to 17 from the Basque Country (Spain). Results showed that sex, age, sexism, and self-esteem predicted teen dating violence and that sex and social problems predicted victimization. Associations between the wide range of variables and types of perpetration and victimization (verbal-emotional, relational, and physical) were also explored. These results could be taken into consideration for future prevention programs.

## 1. Introduction

Teen dating violence (hereafter TDV) refers to a wide range of harmful partner-directed behaviors (psychological, physical, or sexual) among adolescents [1]. Psychological TDV refers to emotionally manipulative acts that try to damage the partner indirectly. Physical TDV include acts such as shoving, slapping, punching, kicking, choking, or burning. Sexual TDV includes sexual activity forced on the partner, ranging from behaviors such as unwanted touching to forced penetration. This study focuses on physical and psychological (verbal-emotional and relational) TDV.

TDV has been on the rise in recent years and is a serious public health problem at the national and international levels [2,3,4,5]. Some research shows that the magnitude of TDV is even greater than violence in adult dating relationships [6,7]. Violence does not appear suddenly in adult relationships, but instead, problems of violence start in adolescence [8].

TDV should be approached from a multicausal perspective, as this is a phenomenon conditioned by the interactive effects both of individual and interpersonal variables [9]. Among the interpersonal factors related to aggression, family factors have received great attention in the scientific literature, such as parenting styles, that have been related to peer violence [10] and bullying or cyberbullying behaviors [11]. These studies show that, when carrying out prevention or intervention programs, the protective or risk effects derived from different parenting styles cannot be forgotten. However, this study will focus on individual factors, as will be justified below.

Various theoretical models have tried to explain violence within the couple. The most widely recognized are the social learning theory, the feminist theory, the power theory, the background/situational model, or the personality/typology model (see the review by Bell and Naugle, 2008). Bell and Naugle [12], in turn, suggest an alternative theoretical conceptualization of intimate partner violence that could be extended to TDV, integrating components of previous theories cohesively, that serves as a theoretical framework for this study. In this conceptualization, Bell and Naugle [12] distinguish antecedents, discriminative stimuli, motivating factors, behavioral repertoire, verbal rules, and consequences related to intimate partner violence. The present study tries to shed more light on the individual behavioral repertoire of TDV perpetrators and victims. Behavioral repertoire refers to socially adaptive skill sets that a person can perform competently under appropriate conditions to successfully attain a desired consequence ([12] p.1104). Adolescents implicated in TDV may have many behavioral repertoire deficits, such as maladjustment characteristics and attitudes (particularly sexist attitudes) as well as deficit in adjustment characteristics (for instance, self-esteem and resilience).

It is also important to take into account a developmental perspective of TDV. Most romantic relationships begin in adolescence. Adolescents’ knowledge about romantic relationships is largely derived from the media and their observation of the relationships of family and friends. Adolescence is characterized by being a stage in which narcissism, the exaggeration of gender-specific roles (males’ control and females’ submission), and the mystification of romance are prevalent, issues that make adolescent relationships especially vulnerable to violence [13]. Dating often leads to increased emotional experiences that may be expressed as aggression towards the partner. This aggression can be physical, verbal, or psychological. Perhaps physical aggression is the most visible type, leading to preventive measures or early intervention. However, there is another form of aggression that frequently appears in teenage love relationships, called relational aggression, which includes verbal or psychological aggression (insults or intimidation). Relational aggression in this study is understood in the same way as by Ellis, Crooks, and Wolfe [14], who define it as behaviors that seek to exercise social control or to harm the relationships between the perpetrator and the victim. This article aims to examine in more depth the implications of this phenomenon and to provide more results that can be included in the different preventive and intervention plans for TDV.

Research with adolescents estimates TDV perpetration rates ranging from 5% to 90% [15,16,17,18,19]. Other studies analyze the perpetration and victimization rates together, reporting ranges from 3% to 70% for victimization [20,21] and between 31% and 50% for perpetration [20,21,22,23]. 

Moreover, TDV occurs both in women and men. Studies in Spain show that males have the highest percentages of perpetration, ranging from 7.5% to 37.8%, compared to 7.1% to 14.9% for females [24,25]. Some authors explain these results considering Spain as a traditionally conservative context, where boys are expected to be rough and dominant and where girls are expected to be submissive and sensitive [26]. However, other studies contradict these results, finding higher rates of perpetration among females [26,27,28]. Fernández-Fuertes, Fuertes, Fernández-Rouco, and Orgaz [29] suggest that gender socialization could be changing, although further research is needed. Concerning victimization, national and international studies appear to indicate a higher victimization rate among females, with rates ranging from 3.6% to 7.8% for males and from 7.9% to 15.5% for females [30,31].

Some authors point to a reduction in aggressive behavior throughout individuals’ life cycle [32,33,34] although the consequences of violence tend to be much more serious at later ages despite being less frequent [35]. Concerning victimization, it appears that younger girls are subject to more aggression than older women [36]. 

The disparity in reported prevalence rates may be due to the different conceptualization of TDV and, specifically, to whether these studies contemplated the typology of violence. Therefore, studies show that verbal (verbal-emotional or psychological) violence is the most common, with rates varying [37,38,39,40,41] between 11% and 90% [34,37,38,39,40,41,42,43]. Likewise, Carver, Joyner, and Udry [44] pointed out that, as age increases, this type of violence increases. Longitudinal studies suggest that, while adolescents can repress their physical aggressive behavior as they grow older, they have serious difficulties to do so with verbal-emotional aggression [21,44,45]. Moreover, verbal-emotional aggression is the most accepted form of violence in youths [46], which makes it more difficult to eliminate. 

On the other hand, other studies [19,37] revealed that a higher percentage of girls in their studies used verbal violence, 95.3% compared to 92.8% in boys. As regards psychological victimization, a rate of 91% was found by previous studies [47]. Taking into account sex, Jackson et al. [7] observed that 81.5% of girls and 76.3% of boys had suffered psychological violence. 

Furthermore, it is very important to analyze the different types of violence, as some studies suggest that relational violence precedes verbal and physical violence [48,49].

As for physical violence, international studies have found perpetration rates of between 10% and 41% [16,19,50]. Concerning sex, some studies show similar rates of violence (25% males and 28% females) [7,51], although others show that females exert the greatest physical violence [19,52]. These last data make us think that TDV is not comparable to gender violence, although it may have some aspects in common. For example, sexist attitudes, which are very present in gender violence, require further study in the area of TDV. Several studies have shown that positive attitudes towards traditional gender roles (both in perpetrators and victims) are related to higher levels of violence in couple relationships [53]. As such, for example, a study [54] found that maintaining traditional gender stereotypes, among others, predicted victimization in dating relationships. Specifically, researches [3] indicated that sexism has an important explicative weight in relational violence.

However, other studies with samples of university students found that the predictive capacity of sexism for the perpetration or victimization of TDV was relatively low, with hostile sexism being the most useful predictor [55]. In the view of some authors [56], hostile sexism is more easily recognized and increasingly socially censured yet benevolent sexism is related to a lower tendency to perceive the inappropriate behavior of others as abusive, when in fact it is. The literature on sexual harassment indicates that more sexist subjects downplay abuse situations, even normalizing them [57], and that such normalization is even higher in those who score higher in benevolent sexism. 

Furthermore, the study of TDV is of great relevance because of its short- and long-term implications. The fact that this type of violence occurs during adolescence aggravates the situation because of the repercussions this can have on the person’s integral development. Thus, previous studies that have tried to identify violence profiles indicate that TDV (both perpetration and victimization) is associated with some risk factors such as higher levels of clinical maladjustment, specifically higher levels of anxiety and depression [22,58,59,60] and difficulties in self-regulation [58,61,62] and social communication [60]. Also, self-esteem is a predictor of TDV, and lower self-esteem has been found both in perpetrators [39,63] and in victims [64,65]. Surprisingly little empirical work exists on the predictive value of self-esteem for TDV, as it seems to make more sense to hypothesize that low self-esteem is a consequence of TVD. Carrascosa et al. [64], for example, noted that a poorer self-concept could be both a risk factor and a consequence of the victimization of TDV, so more studies are required. Moreover, other studies assert that one’s perception of low self-efficacy in interpersonal relationships (self-concept) and one’s tolerance of a partner’s abusive interpersonal style are highly associated [41]. 

According to the U.S. Centers for Disease Control and Prevention [CDC] [1], people who are victims of TDV are more likely (1) to experience symptoms of depression and anxiety; (2) to participate in unhealthy behaviors, such as the use of tobacco, drugs, and alcohol; (3) to exhibit antisocial behaviors, such as lying, stealing, bullying, or hitting; and (4) to think about suicide. In addition, they claim that young people who are victims of TDV in high school are at greater risk of victimization during college.

In this way, it is of great interest to outline the profile of the aggressors and the victims in order to develop adequate prevention and intervention programs. In addition to the variables that could be considered as risk variables, such as clinical mismatches or sexist attitudes, protective variables should be explored. In this sense, the study of resilience could provide an innovative vision in this area of study due to the scarcity of studies on this subject. Resilience research has already been incorporated into other types of violence such as gender violence or child abuse, considering it a high priority factor [66], which could also be applied to TDV. Thus, when relationships are appropriate, they provide security for partners and can foster the development of resilience, but when they are inappropriate or abusive, they can be detrimental to resilience [67]. 

Although in the last years the interest in TDV in the scientific literature has increased, the truth is that the results are still contradictory and leave aspects still to be clarified, such as the differences by sexes or the personal characteristics associated to TDV. Moreover, it is necessary to study the personal profiles not only of the aggressors but also of the victims in order to design effective prevention policies and to be able to decrease the prevalence levels of this phenomenon.

Taking into account all the above, the present study tries to analyze TDV in Spanish adolescents and to explore the relationship between this type of violence (both perpetrated and suffered in the different types: verbal-emotional, relational, and physical) and participants’ sexist attitudes, clinical maladjustment, and resilience. Specifically, taking into account results from previous studies, the following hypotheses were formulated:

(1) We expected to find similar verbal-emotional, relational, and physical TDV rates (perpetration and victimization) in girls and boys [7,16,51,68,69] or, at least, nonsignificant differences, since we consider that TDV is not the same as gender violence, since both girls and boys can be victims and perpetrators.
(2) We expected to find higher TDV rates in older participants [32,34], in part because they are more frequently involved in dating relationships.
(3) TDV was expected to be positively associated with sexism (hostile and benevolent [3]) and with clinical maladjustment [20,22,57] and inversely with resilience. Following the theoretical model by Bell and Naugle [12], these factors could be relevant behavioral repertoire factors related to TDV
(4) Sex, age, sexism, maladjustment, and resilience were all expected to be predictors of TDV.

## 2. Materials and Methods

### 2.1. Participants

The present study involved 268 minors from secondary schools of the Basque Country (Spain), aged between 12 and 17 (*M* = 15.22, *SD* = 1.38), of whom 47% (*n* = 126) were boys and 53% (*n* = 142) were girls. Most of them came from the Basque Country (89.6% (*n* = 240)), 7.8% (*n* = 21) were of foreign origin, and 2.6% (*n* = 7) were from the other communities of Spain. Of the participants in the study, 72% (*n* = 193) came from concerted schools and 28% (*n* = 75) came from public schools. 

### 2.2. Instruments

*Conflict in Adolescent Dating Relationships Inventory* (CADRI; [70]). The present study was carried out using the Spanish version [39]. Specifically, the questionnaire consists of 17 items that analyze the different types of perpetration of violence: perpetration of relational violence (e.g., “I said things to his/her friends about him/her to make them go against him/her”), perpetration of verbal-emotional violence (e.g., “I brought up in conversation something bad that he/she had done in the past”), and perpetration of physical violence (e.g., “I pushed him/her or I shook him/her”). In addition, another 17 items measure victimization: relational victimization (e.g., “He/she tried to separate me from my group of friends”), verbal-emotional victimization (e.g., “He/she insulted me with humiliation”), and physical victimization (e.g., “He/she threw something at me”). Adolescents are asked to identify how often they have experienced these situations in their romantic relationship: Never (this has not happened in our relationship), rarely (1 or 2 times), sometimes (between 3 and 5 times), or frequently (6 or more times). In the present study, the reliability coefficients (Cronbach’s alpha) for each subscale of perpetration (relational, verbal-emotional, and physical) were 0.65, 0.81, and 0.80, respectively. The reliability of the victimization subscale (relational, verbal-emotional, and physical) was 0.64, 0.87, and 0.82, respectively. For the total scale of perpetration, it was 0.72, and for the total scale of victimization, it was 0.78. The total alpha coefficient for this sample was 0.80.

*Behavior Assessment System for Children and Adolescents (BASC-S3*; [71]. The *BASC-S3* personality self-report is an inventory consisting of 185 statements with dichotomous responses (true/false), grouped into 14 scales. These 14 scales are grouped into three overall dimensions: personal adjustment, clinical maladjustment, and school maladjustment. In this study, clinical maladjustment and personal adjustment dimensions were assessed. The clinical maladjustment scales are negative attitude toward school, negative attitude toward teachers, sensation seeking, atypicality, locus of control, somatization, social stress, anxiety, depression, and sense of inadequacy. The personal adjustment dimension consists of interpersonal relationships, relationships with parents, self-esteem, and self-confidence. Cronbach’s alpha for the personal adjustment dimension was 0.77 and, for the clinical maladjustment dimension, was 0.76. The internal consistency (Cronbach’s alpha) of the total scale was 0.80.

*Ambivalent Sexism Inventory for Adolescents (ASI_A*; [72]). This is a 20-item instrument, rated on a 6-point Likert-type scale, ranging from 1 (strongly disagree) to 6 (strongly agree). The *ASI_A* is an adaptation of the *ASI* [72] for the adolescent population, which provides one measure of hostile sexism and another measure of benevolent sexism. Hostile sexism, the classic sexism, refers to prejudice attitudes or discriminatory behaviors that are based on women’s inferiority. It is assessed through 10 items composed of three subscales: protective paternalism: “Boys should exert control over who their girlfriends interact with”; complementary gender differentiation: “Sometimes girls use the fact of being ‘girls’ to say they should be treated in a special way”; or heterosexual intimacy: “Girls are too easily offended”. Benevolent sexism refers to a whole set of sexist attitudes towards women in which they are stereotyped and limited to certain roles but using a positive tone towards them, especially in social gatherings and when seeking for intimacy. This dimension also consists of 10 items, divided into three subscales: protective paternalism: “Girls should be cherished and protected by boys” or “Boys should take care of girls”; complementary gender differentiation: “Girls, compared to boys, tend to be more sensitive to others’ feelings”; and heterosexual intimacy: “Romantic relationships are essential for achieving true happiness in life”. In the sample studied, the reliability index (Cronbach’s alpha) of the scale was α = 0.80 (hostile sexism α = 0.67 and benevolent sexism α = 0.77).

*Resilience Scale (CD-RISC*; [73]; Spanish adaptation [74]). The questionnaire is intended for adolescents and adults. The instrument consists of 25 items (e.g., “I can do my best to accomplish anything”; “I can achieve my goals”; or “I know how to seek help when I need it”), presented on a 5-point Likert ranging from 1 (completely false) to 5 (almost always true). The test consists of the following dimensions: (1) persistence-tenacity-autonomy (personal perception of effectiveness); (2) control under pressure (ability to protect one’s integrity); (3) adaptability and support networks (family as support network); (4) control and purpose (determining existential purposes and quality of control); and (5) spirituality (understood as the search for meaning). The Cronbach alphas for each dimension are as follows: persistence-tenacity-autonomy (α = 0.76), control under pressure (α = 0.65), adaptability and support networks (α = 0.60), control and purpose (α = 0.59), and spirituality (α = 0.57). The Cronbach alpha for the total scale was 0.86.

*Children’s Depression Scale (CDS*; [75]), Spanish adaptation [76]. This contains 66 items, 48 of a depressive type and 18 of a positive type. It is rated on a 5-point Likert-type scale, ranging from 1 (strongly agree) to 5 (strongly disagree). These two sets of items are grouped into two general independent subscales: total depressive (e.g., “I often think nobody cares for me”) and total positive (e.g., “I get fun out of the things I do”). The total depressive scale consists of six subscales: affective response, social problems, self-esteem, preoccupation with sickness and death, guilt, and miscellaneous depression (includes types of depressive issues that could not be grouped together to form a single entity). The total positive scale contains two subscales: pleasure and enjoyment, and miscellaneous positive items (includes positive aspects that could not be brought together to form an entity and whose absence may lead to important depressive manifestations in the child or adolescent). In this study, the internal consistency of the total depressive dimension (α = 0.70) and the total positive (α = 0.80) was good.

### 2.3. Procedure

The project was approved by the Ethics Committee of the University of the Basque Country (UPV/EHU), (M10/2016/158) and obtained a predoctoral scholarship (PIF16/257) from the UPV/EHU. The year of data collection was between 2017 and 2018.

To carry out the selection of the educational centers, random discrimination was made among all the schools (public, subsidized, and private) in the Basque Autonomous Community. All the schools registered in the database of the Department of Education of the Basque Government were introduced, and the schools were chosen employing a random formula. The researchers presented and delivered the research report to the directors of each school so that they could approve the research and sign the research agreement. Both the participants and their families or legal guardians signed a document of consent which is kept in the file registered for this purpose at the University of the Basque Country (UPV/EHU). The consents for the relatives and/or legal guardians were collected with the help of the directors of the schools. Family members received information about the investigation by email and letter, emphasizing that participation in the study was voluntary. The students received the email from the guidance department of the school and were priorly informed by the researcher and again on the day of the sample collection. A total of 18 students were excluded from the sample because they did not bring family consent on the day of sample collection.

### 2.4. Data Analyses

The statistical analyses, aimed at achieving the objectives, were tested using IBM SPSS Statistics for Windows, version 25 (IBM Corp., Armonk, N.Y., USA)). Two assumptions of normality and homogeneity of variations were then demonstrated prior to the corresponding analysis to decide whether parametric or nonparametric tests were used. Specifically, the critical level of *p* < 0.05 of the Kolmogorov–Smirnov statistic was analyzed to determine the distribution of data for the analysis of group differences. Descriptive analyses were performed using sociodemographic variables. We used the chi-square test and Cramer’s coefficient V for effect size to verify associations between types of victimization and perpetration of violence in relationships and sociodemographic variables. We highlight that the different dimensions of TDV and victimization of the CADRI as well as the total scores of both dimensions were dichotomized to categorize the groups (0 = *no* and 1 = *yes*). Adolescents were classified as perpetrators or victims of TDV if they marked one or more elements of perpetration/victimization. The same method was applied for each subscale of perpetration and victimization of TVD. Subsequently, we looked for differences in personal adjustment, clinical maladjustment, resistance, and sexist attitudes between groups of perpetrators and victims of violence with the nonparametric Mann–Whitney test, as the data did not present a normal distribution, considering the categorization of those who perpetrated and suffered from TDV and of those who did not perpetrate or suffer from TDV. Finally, based on the previous analysis, we tried to identify the predictive variables in this group in general and in this phenomenon (teen dating violence) in particular, through binary logistic regressions. However, examining Table 1, we confirmed the scarcity of students who had perpetrated relational violence (*n* = 25) and those who had perpetrated physical violence (*n* = 13). At the crosstabs, in the case of victimization, there was also a scarcity of cases (*n* = 1 for relational violence and *n* = 1 for physical violence).

## 3. Results

### 3.1. The Victimization and Perpetration of Teen Dating Violence and Their Types by Gender and Age

A total of 29.1% (*n* = 78) adolescents reported having been violent in their relationships (perpetration) in the last twelve months, and 36.2% (*n* = 97) said they had experienced violent situations (victimization). 

Concerning sex, 15.4% of the girls and 84.6% of the boys had perpetrated violence in their dating relationships. In terms of victimization, the girls reported the highest rates (87.6%) in comparison to the boys (12.4%). The two results show statistically significant associations with a moderate to large effect size, χ^2^ = 62.44, *p* < 0.01, Cramer’s V = 0.48, in the perpetration of teen dating violence, and χ^2^ = 73.24, *p* < 0.01, Cramer’s V = 0.52, in victimization. 

As for age, in relation to perpetration, 75.6% of the sample were adolescents between 15 and 17 years old and 24.4% were between 12 and 14 years old, χ^2^= 7.90, *p* < 0.01, Cramer’s V = 0.17. Likewise, in relation to victimization, 80.4% belonged to the oldest group (15–17) and 19.6% belonged to the youngest (12–14), χ^2^ = 20.42, *p* < 0.01, Cramer’s V = 0.28. In this case, the associations, although statistically significant, indicated a smaller effect size than that found with sex.

Table 1 shows the associations between the types of victimization and perpetration of violence (relational, verbal-emotional, and physical) in partner relationships by sex and age. The largest association both for victimization and perpetration was in the verbal-emotional type, showing statistically significant associations, with an intermediate effect size.

Concerning age, the associations with verbal-emotional perpetration were significant because 77.8% (*n* = 56) of the students were between 15–17 years old and 22.2% (*n* = 16) were between 12–14 years old. For verbal-emotional victimization, the associations with age showed intermediate effect sizes, as 82.4% (*n* = 75) of the students were in the 15–17 age range and 17.6% (*n* = 16) were between 12–14 years. No other significant associations were found.

### 3.2. Variables Associated with the Victimization and Perpetration of Violence in Dating Relationships

When analyzing the group differences in the scales used in this study, the adolescents who perpetrated the violence showed the highest scores in hostile sexism (total hostile sexism, *U* = 5559.00, *z* = 3.22, *p* < 0.01, *r* = 0.20) and its subscales: protective paternalism, complementary gender differentiation, and heterosexual intimacy. Total benevolent sexism also showed higher scores, *U* = 5223.50, *z* = 3.80, *p* < 0.01, *r* = 0.23) than its subscales: protective paternalism and heterosexual intimacy. As can be seen, the effect sizes vary from small to moderate (See Table 2). 

In relation to the types of violence perpetrated in couple relationships, statistically significant differences were found between relational violence and benevolent sexist attitudes, the one that had the greatest effect was heterosexual intimacy, followed by total benevolent sexism and protective paternalism. In addition, there was also a significant difference with one of the resistance subscales: control under pressure. All adolescents who perpetrated relational violence showed higher scores on the sexism subscales and lower scores on the control under pressure subscale (See Table 3).

As for verbal-emotional violence, the perpetrators had lower self-esteem scores with a small effect size. Regarding sexist attitudes, the medians were higher among respondents who perpetrated verbal-emotional violence (total hostile sexism and total benevolent sexism, in the following subscales: hostile sexism—protective paternalism, hostile sexism—complementary gender differentiation, benevolent sexism—protective paternalism, and benevolent sexism—heterosexual intimacy (See Table 4).

Finally, physical violence showed statistically significant differences with social stress, anxiety, benevolent sexism—protective paternalism, and benevolent sexism—complementary gender differentiation, all with small effect sizes. That is, all youth who perpetrated physical violence reported greater social stress, greater anxiety, and higher rates of benevolent paternalistic sexism and gender-differentiated sexism (See Table 5).

In the case of total victimization (relational, verbal-emotional, and physical) the adolescents in the study showed statistically significant differences in the social problems subscale, *U* = 6845.00, *z* = 2.38, *p* < 0.05, *r* = 0.14 (no: *Mdn* = 2 (1–4.5) and yes *Mdn* = 2.3 (1–4.9)). In other words, those who admitted suffering victimization obtained higher medians in social problems than the rest of the adolescents, who reported not suffering any violence by their dating partners. 

For relational victimization, results only revealed statistically significant differences in resilience, specifically in the subscale of control under pressure, *U* = 2668.00, *z* = 2.26, *p* < 0.05, *r* = 0.14 (no: *Mdn* = 24 (13–33) and yes *Mdn* = 23 (14–31)), where those who suffered relational violence obtained lower medians. 

In the case of verbal-emotional victimization, social problems again revealed statistical differences, *U* = 6803.00, *z* = 2.16, *p* < 0.05, *r* = 0.13 (no: *Mdn* = 2 (1–4.5) and yes *Mdn* = 2.3 (1–4.8)), with those reporting verbal-emotional victimization obtaining a higher median. Also, those who suffered verbal-emotional victimization obtained higher scores in hostile sexism—heterosexual intimacy, *U* = 6729.50, *z* = 2.21, *p* < 0.05, *r* = 0.14 (no: *Mdn* = 6 (3–16) and yes *Mdn* = 7 (3–17)), in benevolent sexism—total benevolent sexism, *U* = 6834.00, *z* = 2.03, *p* < 0.05, *r* = 0.12 (no: *Mdn* = 22 (10–51) and yes *Mdn* = 25 (10–57)), and in benevolent sexism—complementary gender differentiation, *U* = 6445.00, *z* = 2.69, *p* < 0.05, *r* = 0.16 (no: *Mdn* = 6 (3–15) and yes *Mdn* = 7 (3–17)), with small effect sizes. 

Finally, in the case of physical victimization, the locus of control, *U* = 785.50, *z* = 2.11, *p* < 0.05, *r* = 0.13 (no: *Mdn* = 3 (1–13) and yes *Mdn* = 5 (2–13)) and control under pressure, *U* = 766.50, *z* = 2.27, *p* < 0.05, *r* = 0.14 (no: *Mdn* = 23 (14–33) and yes *Mdn* = 19 (25.5–30)) subscales showed statistically significant differences. Adolescents who reported physical victimization reported higher scores in the external locus of control and lower scores in control under pressure. 

### 3.3. Predictive Factors of Victimization and Violence in Dating Relationships in Adolescents in School

Logistic binary regressions were performed to determine the factors associated with victimization and perpetration of violence in dating relationships. The models were tested for verbal-emotional violence and victimization as well as for the total scores because the prevalence rates were higher. Table 6 shows two multivariate logistic regression models for perpetration: total score violence and verbal-relational violence perpetration. 

Model 1 is the predictive model for total violence, of which the results show that being older (15–17) increased by 2.78 the chances of perpetration of violence (95% confidence interval (CI) [1.38, 5.58]) and that, compared to girls, boys were 14.96 times more likely to perpetrate violence (95% CI [6.85, 31.16]). Self-esteem had an inverse relationship, with higher levels of self-esteem decreasing the chances of the adolescents committing violence in their relationships, odds ratio (OR) = 0.80, 95% CI [0.68, 0.94]. Benevolent sexism was associated with a greater likelihood of perpetrating violence, OR = 1.07, 95% CI [1.01, 1.13]. 

Model 2 is the predictive model of verbal-emotional violence. Results show that being male, OR = 16.47, 95% CI [7.58, 35.78], and older (15–17 years), OR = 3.77, 95% CI [1.85, 7.69], increased the likelihood of committing verbal-emotional violence against the dating partner. On the other hand, the increase of one unit in self-esteem reduced by 0.85 the probability of exerting verbal-emotional violence (95% CI [0.72, 0.99]).

Table 7 presents the patterns of victimization. Model 3 shows the predictive model of total victimization, where girls obtained very high ORs, increasing by 17.98 times (95% CI [8.59, 37.62]) the possibilities of victimization in dating relationships. Social problems scores also were associated with the probability (OR = 1.50, 95% CI [1.02, 2.20]) of suffering victimization. 

Model 4 is the predictive model of verbal-emotional victimization. Once again, being female increased by 19.90 (95% CI [9.13, 43.36]) the probability of suffering verbal-emotional victimization. Moreover, the 12–14-year-old age group showed a reduced likelihood (OR = 0.15, 95% CI [0.07, 0.32]) of suffering this type of victimization. On the contrary, having social problems increased by 1.39 the probability of suffering verbal-emotional violence (95% CI [1.04, 2.05]). 

## 4. Discussion

Violence in teenage relationships is still a serious social problem. With the aim of examining this topic in greater depth, two objectives were established in this study: (1) to analyze TDV in Spanish adolescents in school, expecting to find similar rates of TDV (perpetration and victimization)—verbal-emotional, relational, and physical—in girls and boys of the sample and higher rates in older participants and (2) to explore the relationship between this kind of violence (both perpetrated and suffered, in its various types: verbal-emotional, relational, and physical) and participants’ sexist attitudes, clinical maladjustment, and resilience. TDV was expected to be positively associated with sexism (hostile and benevolent) and clinical maladjustment and inversely associated with resilience; also, sex, age, sexism, maladjustment, and resilience were expected to be predictors of TDV. 

Concerning the first objective, the results showed that 29.1% of the participants had perpetrated violence in their relationships in the past twelve months and that 36.2% claimed to have suffered violent situations. These percentages are within the range observed in other studies [17,58,68,77]. Considering sex, it was observed that boys had higher rates of perpetration and girls had higher rates of victimization. In this sense, some studies corroborate these results [24,25], but there are also studies that claim that girls perpetrate more violence than boys [26,27,28].

As far as perpetration rates are concerned, verbal-emotional scores were the highest (26.9% vs. 9.3% of relational and 4.9% of physical violence). In addition, all types of DV perpetration showed statistically significant associations with sex, with boys obtaining higher percentages than girls. These results are similar to those observed in many studies [7,16,34,37,38,41,45]. However, other studies [19,52] found that girls exerted the most physical violence. Perhaps, these discrepant results concerning sex could be explained by the use of different measuring tools or the different social contexts in which the studies were conducted. 

In victimization, verbal-emotional victimization again revealed the highest scores (34% vs. 11.2% for relational and 3.7% for physical violence). Statistically significant associations were also found in sex-based victimization rates, but the girls showed higher percentages than the boys in all types of victimization, in the same vein as other studies [30,31].

The higher percentage of boys in all types of perpetration and of girls in all types of victimization could also be related to the conservative sociocultural context described by other authors [78] and to the so-called “intimate terrorism” by Johnson [79], by which violence is embedded in a relationship context of general coercive control perpetrated primarily by boys and rooted in patriarchal attitudes. This interpretation is supported by the results obtained in the present study on the role of sexism in TDV. 

The results also show that older participants (75.6%, 15–17-year-olds) engaged in more violent behaviors when compared to younger ones (24.4%, 12–14-year-olds), with verbal-emotional violence being the most widely used in the older adolescents (77% vs. 22%). Regarding victimization, these data contradict studies that observed a reduction in aggressive behaviors throughout the lifetime [32,33], but this could be explained by the participants’ older age or the different cultures in which those studies were conducted. In addition, older participants (15–17 years) had higher scores in victimization (80.9% in 15–17 year-olds vs. 19.6% in 12–14 year-olds) in the same vein as other studies [36,78] showing higher verbal-emotional victimization at older ages (82.4% in 15–17 year-olds vs. 17.6% in 12–14 year-olds). 

The results show that TDV is associated with sexism, both benevolent and hostile. Thus, participants who claimed to have been violent towards their partners reported higher levels of sexism than those who had never resorted to any form of violence in their relationships. Specifically, verbal-emotional TDV perpetration shows significant differences with hostile sexism (which is better known as “sexism” in society and is more criticized and disapproved), whereas physical and relational violence show significant differences with benevolent sexism. In fact, benevolent sexism was identified as a predictor of TDV perpetration. Benevolent sexism is considered more subtle and is not so disapproved because it is disguised with a more positive tone, but it is still sexist and, as has been shown, is associated with relational violence (i.e., trying to separate one’s partner from their network of friends and family) and with physical violence. As some authors [56] pointed out, this kind of sexism prevents one from realizing that certain attitudes are indeed sexist. 

Regarding victimization, it is striking that sexism (hostile and benevolent) reveals statistically significant differences between the kinds of verbal-emotional victimization and not in the other types of victimization (physical or relational). That is, people who are victims of verbal-emotional TDV also score higher in sexism. Thus, intervention to combat sexism is interesting not only for the prevention of perpetration of violence but also for the prevention of victimization in teen dating relations. However, it is important to address sexism taking sex into account, as it is a predictor of TDV. Considering the analysis of sexism as a function of sex could be enriching and could help to understand why it occurs more in one sex than the other. In the case of the boys, many of them may feel pressured by masculine ideals, the ideals that restrict emotional expressions and socially drive expectations of domination and aggression, encouraging boys to participate in violent acts such as intimidation and verbal and/or physical aggression [47]. Also, it will help to have a broader view of gender roles and, therefore, of sexism, not considered as biological representations but as a set of psychological and socially constructed ideas that can, therefore, be changed [80].

With regard to the adjustment/ maladjustment of TDV perpetrators or victims (compared to those who are not), the results are interesting: on the one hand, participants who reported resorting to relational violence with their partners scored lower in control under pressure, a subscale of resilience that indicates emotion regulation in the face of stressful situations. Control difficulties are evident in those who resort to violence as a form of conflict resolution, so these results would be consistent with our expectations. On the other hand, people who resort to verbal-emotional violence scored lower in self-esteem than those who do not use this form of violence with their partners. Finally, people who resort to physical violence scored higher in anxiety and social stress (feeling stress in their social relationships). Although previous studies had already indicated the relationship between TDV and adjustment problems in the perpetrators (anxiety, depression, low self-esteem, etc.) [22,58,59,64], this study provides more detailed information on the types of violence, which could have practical implications for prevention and intervention policies. For example, emotional aspects (such as anxiety and stress) and aspects of emotion regulation (such as pressure control) have shown significant differences depending on the types of TDV. 

The fact that low self-esteem is a predictor of TDV perpetration (both total and verbal-emotional) provides relevant information about adolescents who are violent towards their partners: although they seem to be strong and controlling, with an attitude of imposition towards their partners, this facade only hides their low self-esteem. This would, therefore, be an important aspect to address with these teenagers.

Concerning victims’ adjustment/maladjustment, the results show that those who reported verbal-emotional victimization had more social problems, which could indicate that these people, in addition to becoming involved in conflictive couple relationships, also have problems in their social relationships in general. In fact, social problems are one of the predictors of victimization in this study. Given the importance of peer relations at these ages, all of this could be indicative of significant personal adjustment problems in these adolescents, which may make them more vulnerable to engaging in conflictive couple relationships, with significant negative consequences for their psychosocial development.

On the other hand, people who reported relational and physical victimization scored lower in control under pressure, which is a characteristic of resilient people; that is, as expected, these people who were victimized showed less resilience.

Finally, it should be noted that adolescents who reported physical victimization showed higher external locus of control (compared to those who did not suffer victimization), which makes them feel that they have no control over what goes on in their lives, leading to hopelessness, which could be associated with the anxious-depressive symptoms described in previous studies [22,58,59,60] among adolescents, with verbal-emotional violence being the most recurrent. In addition, in the three types of violence analyzed (verbal-emotional, relational, and physical), older boys carried out the most violent behaviors and older girls suffered the most victimization. 

It is confirmed that violence and victimization in couple relationships can be relational, verbal-emotional, and physical and that all three are present among teenage couples in our environment and must be taken into consideration. Thus, relational and verbal-emotional violence, being more subtle, can go unnoticed, and precisely for this reason, they should be particularly relevant in prevention and intervention programs. 

These results highlight that, despite the national policies against gender-based violence, the high rates of perpetration and victimization in dating relations are troubling, preventing neither males from responding to conflicts with more violent behavior nor females from suffering it. In addition, it has been observed that, in later adolescence, both perpetration and victimization increase. This situation requires further analysis of why these behaviors increase in later adolescence, what happens so that adolescents respond more aggressively to conflicts, and why most of the victims are female. In addition, it should not be forgotten that, currently, this is the same pattern that is repeated in adulthood. No doubt, we must continue to delve into the problem, to analyze the most successful actions and policies, and to continue to evaluate their effectiveness according to age, sex, culture, and contexts (family, school, social, etc.), among others. The results, therefore, suggest the importance of developing training campaigns targeting educational professionals and society itself to eradicate TDV, enhancing egalitarian relations, and of developing psychoeducational intervention programs that promote respect for human rights, to address emotional aspects (emotion regulation, self-esteem, peer relationships, etc.), and to prevent violence at younger ages. Future studies could explore the importance of emotional intelligence in this type of violence and the possible incorporation of emotional education as a way of preventing these violent behaviors among adolescents. In addition, adolescents’ training and the awareness raising of sexism types should be considered a key aspect in the prevention of TDV.

With regard to the limitations of this work, one of them is the cross-sectional design of the study, as it provides data during a specific period of time in a given sample. Another limitation could be the instrument battery used for data analysis. Specifically, the information was collected through self-reports, which may also introduce some bias in the investigation. It might be interesting to replicate this same study with other tools and strategies for information collection, such as hetero-reports and structured or semi-structured interviews. 

In the future, it would be interesting to carry out a longitudinal design in the study of TDV, to analyze the continuity over time of this type of violence, and to determine whether the perpetration and victimization of TDV increases or decreases at more advanced ages among youth (18 to 23 years), that is, to assess whether the variables that were revealed as significant in this study are maintained at more advanced ages because some studies support that violent relationships decrease in adulthood.

Besides, it should be borne in mind that social networks are currently the most important means of communication for young people. Studies show that the use of technology in romantic relationships is frequent [80,81,82,83] and that it increases their capacity to control the partner at all times, leading to a more violent relationship [81]. Through phone apps (such as WhatsApp) or social media (such as Facebook), young people control their partners, producing more discord and violence between them. Prevention and intervention programs will therefore also need to take into account these new forms of relationship offered by the new technologies.

## 5. Conclusions

Teen Dating violence deserves special attention in social science research, as there are still too many unresolved questions and inconsistencies between the different studies. It should not be treated in the same way as violence in adult couples, since, although it surely shares similarities, the fact that it occurs among adolescents makes it different. This is due, on the one hand, to the effects it can have on adolescents, because of the developmental period, and on the other hand, to the effect it can have on the future affective relationships of these young people. Thus, although there are multiple aspects involved in this type of violence, research on the associated factors is necessary. The present study focuses on the personal aspects, both of victims and of perpetrators of violence, and it is observed that factors such as sexism, self-esteem and social problems play a relevant role. All of them are factors that can be worked on from an early age in schools, families, the media... Therefore, we all, as part of this society, have the responsibility to intervene to prevent this type of violence from its beginnings.

## Figures and Tables

**Table 1:** Association between types of violence and victimization, and sociodemographic variables.

	Total and Types of Perpetration of Violence				Total and Types of Victimization			
	Total Violence *n* (%)	Relational *n* (%)	Verbal-Emotional *n* (%)	Physical *n* (%)	Total Victimization *n* (%)	Relational *n* (%)	Verbal-Emotional *n* (%)	Physical *n* (%)
Sex Boys	Yes 66 (52.4%) No 60 (47.6%)	Yes 22 (17.5%) No 104 (82.5%)	Yes 64 (50.8%) No 62 (49.2%)	Yes 10 (7.9%) No 116 (92.1%)	Yes 12 (9.5%) No 114 (90.5%)	Yes 1 (0.8%) No 125 (99.2%)	Yes 10 (7.9%) No 116 (92.1%)	Yes 1 (0.8%) No 125 (99.2%)
Girls	Yes 12 (8.5%) No 130 (91.5%)	Yes 3 (2.1%) No 139 (97.9%)	Yes 10 (7%) No 132 (93%)	Yes 3 (2.1%) No 139 (97.9%)	Yes 85 (59.9%) No 57 (40.1%)	Yes 29 (20.4%) No 113 (79.6%)	Yes 81 (57%) No 61 (43%)	Yes 9 (6.3%) No 113 (93.7%)
χ2	62.44 *	18.59 **	60.41 **	4.91 *	73.24 **	25.86 **	71.79 **	5.71 *
*V* *	0.48	0.26	0.48	0.14	0.52	0.31	0.52	0.15
Age 12–14	Yes 19 (19%) No 81 (81%)	Yes 8 (8%) No 92 (92%)	Yes 16 (16%) No 84 (84%)	Yes 4 (4%) No 96 (96%)	Yes 19 (19%) No 81 (81%)	Yes 8 (8%) No 92 (92%)	Yes 16 (16%) No 84 (84%)	Yes 4 (4%) No 96 (96%)
15–17	Yes 59 (35.1%) No 109 (64.9%)	Yes 17 (10.1%) No 151 (89.9%)	Yes 56 (33.3%) No 112 (66.7%)	Yes 9 (5.4%) No 159 (94.6%)	Yes 78 (46.4%) No 90 (53.6%)	Yes 22 (13.1%) No 146 (86.9%)	Yes 75 (44.6%) No 93 (55.4%)	Yes 6 (3.6%) No 162 (96.4%)
χ2	7.90 *	0.33	9.59 *	0.25	20.42 **	1.64	22.93 **	0.032
*V* *	0.17	0.04	0.19	0.03	0.28	0.08	0.29	0.011

Note: ** *p* < 0.01; * *p* < 0.05; V = Cramer’s V; strength of association.

**Table 2:** Significant results for variables associated to perpetration of violence in teen dating relationships.

			Total Hostile Sexism	H.Protective Paternalism	H.Gender Differentiation	H.Heterosexual Intimacy	Total Benevolent Sexism	B.Protective Paternalism	B.Heterosexual Intimacy
Total violence	No	Mdn (IIQ)	20 (10–49)	6 (4–19)	7 (3–18)	6 (3–16)	22 (10–51)	4 (9–21)	6 (3–18)
	Yes	Mdn (IIQ)	27 (24–51)	7 (4–20)	8.5 (3–17)	8 (3–17)	27 (10–57)	12 (4–22)	8 (3–7)
	*U*		5559.00 **	6290.50 **	5844.50 **	5882.00 **	5223.50 **	5423.00 **	5643.00 **
	*z*		3.22	2.84	2.73	2.66	3.80	3.46	3.09
	*r*		0.20	0.17	0.17	0.16	0.23	0.21	0.19

Note: ** *p* < 0.01. * *p* < 0.05. r = Effect size; H = Hostile Sexism; B = Benevolent Sexism.

**Table 3:** Significant results for variables associated to perpetration of relational violence in teen dating relationships.

			Control under Pressure	Total Benevolent Sexism	Protective Paternalism	Heterosexual Intimacy
**Relational violence**	No	Mdn (IIQ)	24 (14–22)	23 (10–57)	10 (4–22)	6 (3–18)
	Yes	Mdn (IIQ)	22 (15–27)	31 (10–45)	13 (4–20)	9 (3–17)
	*U*		2230.50 *	2008.00 *	2195.50 *	1847.50 **
	*z*		2.19	2.79	2.28	3.24
	*r*		0.13	0.17	0.14	0.20

Note: ** *p* < 0.01. * *p* < 0.05; r = Effect size; H = Hostile Sexism.

**Table 4:** Significant results for variables associated to perpetration of verbal-emotional violence in teen dating relationships.

			Self-Esteem	Total Hostile Sexism	H.Protective Paternalism	H.Gender Differentiation	Total Benevolent Sexism	B.Protective Paternalism	B.Heterosexual Intimacy
Verbal-emotional violence	No	Mdn (IIQ)	7 (1–8)	6 (4–8)	6 (4–19)	7 (4–10)	22 (10–51)	9 (4–21)	6 (3–18)
	Yes	Mdn (IIQ)	6 (1–8)	6 (4–8)	7 (4–20)	7 (4–10)	27.50 (10–57)	12 (4–22)	8 (3–18)
	*U*		25744.00 **	5723.00 *	5890.00 *	5674.00 *	4897.00 **	5052.00 *	5178.00 **
	*z*		2.60	1.91	1.92	1.97	2.79	2.01	2.70
	*r*		0.13	0.12	0.16	0.12	0.27	0.12	0.17

Note: ** *p* < 0.01. * *p* < 0.05; r = Effect size; H = Hostile Sexism; B= Benevolent Sexism.

**Table 5:** Significant results for variables associated to perpetration of physical violence in teen dating relationships.

			Social Stress	Anxiety	B.Protective Paternalism	B.Gender Differentiation
**Physical violence**	No	Mdn (IIQ)	3 (1–13)	8 (1–14)	10 (4–22)	6 (3–16)
	Yes	Mdn (IIQ)	5 (1–10)	10 (2–12)	17 (4–22)	10 (3–17)
	*U*		925.50 **	1027.50 *	872.50 *	967.00 *
	*z*		2.71	2.32	2.01	2.02
	*r*		0.17	0.14	0.12	0.12

Note: ** *p* < 0.01. * *p* < 0.05. r = Effect size; H = Hostile Sexism; B = Benevolent Sexism.

**Table 6:** Variables predicting violence in dating relationships.

Model 1—Total Violence (*N* = 268)						
Variable	B	SE	Wald	*p*	OR	95% CI
Constant	−1.13	0.95	1.37	0.241	0.328	
Sex (ref: girl)	2.70	0.40	46.11	0.00	14.96	[6.85, 31.16]
Age (ref: 12–14 age)	1.02	0.356	8.21	0.04	2.78	[1.38, 5.58]
BASC_S3_Self-esteem	−0.22	0.085	6.64	0.10	0.80	[0.68, 0.94]
ISA_ Benevolent sexism	0.07	0.29	5.37	0.20	1.07	[1.01, 1.13]
**Model 2—Verbal-Emotional Violence (*N* = 268)**						
Constant	−2.60	0.65	16.14	0.00	0.07	
Sex (ref: girl)	2.80	0.40	50.11	0.00	16.47	[7.58, 35.78]
Age (ref: 12–14 age)	1.33	0.36	13.38	0.00	3.77	[1.85, 7.69]
BASC_S3_Self-esteem	−0.17	0.09	3.81	0.05	0.85	[0.72, 0.99]

Note: Model 1: R^2^ = 0.90 (Hosmer–Lemeshow), 0.27 (Cox–Snell), 0.39 (Nagelkerke). Note: Model 2: R^2^ = 0.66 (Hosmer–Lemeshow), 0.17 (Cox–Snell), 0.29 (Nagelkerke).

**Table 7:** Variables predicting victimization in dating relationships.

Model 3—Total Victimization (*N* = 268)						
Variable	B	SE	Wald	*p*	OR	95% IC
Constant	−4.43	0.648	43.70	0.00	0.012	
Sex (ref: boy)	2.90	0.377	58.79	0.00	17.98	[8.59, 37.62]
CDS-Social problems	0.402	1.97	4.18	0.41	1.50	[1.02, 2.20]
**Model 4—Verbal-Emotional Victimization (*N* = 268)**						
Constant	−2.73	0.56	24.16	0.00	0.06	
Sex (ref: boy)	2.99	0.40	56.60	0.00	19.90	[9.13, 43.36]
Age (ref: 15–17 age)	1.87	0.37	25.84	0.00	0.15	[0.07, 0.32]
CDS-Social Problems	0.33	0.20	2.77	0.10	1.39	[1.04, 2.05]

Note: Model 3: R^2^ = 0.94 (Hosmer–Lemeshow), 0.7 (Cox–Snell), 0.37 (Nagelkerke); Note: Model 4: R^2^ = 0.74 (Hosmer–Lemeshow), 0.17 (Cox–Snell), 0.27 (Nagelkerke).
